# The clinical value of lymphocyte percentages and the monocyte-to-lymphocyte ratio in differentiating immune-mediated necrotizing myopathy from dermatomyositis

**DOI:** 10.3389/fneur.2025.1581206

**Published:** 2025-05-26

**Authors:** Xue Ma, Kaikai Huo, Huajie Gao, Huizhen Ge

**Affiliations:** ^1^Department of Neurology, The First Affiliated Hospital of Xi’an Jiao Tong University, Xi’an, China; ^2^Department of Pulmonary and Critical Care Medicine, The Second Affiliated Hospital of Xi’an Jiaotong University, Xi’an, China; ^3^Department of Neurology, Tongji Hospital, Tongji Medical College, Huazhong University of Science and Technology, Wuhan, China

**Keywords:** immune-mediated necrotizing myopathy, dermatomyositis, monocyte-to-lymphocyte ratio, percentages of lymphocytes, red blood cell distribution width

## Abstract

**Objective:**

Immune-mediated necrotizing myopathy (IMNM) and dermatomyositis (DM) represent distinct subtypes of idiopathic inflammatory myopathies (IIMs). While both conditions share clinical manifestations, including muscle weakness and inflammatory infiltrates on muscle biopsy, their pathophysiological characteristics differ significantly. This study investigated the clinical utility of hematological inflammatory biomarkers in differentiating these two entities.

**Methods:**

In this retrospective analysis, we compared complete blood count parameters among 27 patients with IMNM, 14 patients with DM, and 85 healthy controls (HC). Demographic characteristics, clinical presentations, and hematological indices including the neutrophil-to-lymphocyte ratio (NLR), monocyte-to-lymphocyte ratio (MLR), and platelet-to-lymphocyte ratio (PLR) were analyzed.

**Results:**

Myalgia and skin rash were observed more frequently in the DM group compared to the IMNM group. The patients with IMNM exhibited significantly higher serum creatine kinase (CK) and lactate d0.2815ehydrogenase levels. Red blood cell distribution width (RDW), monocyte counts, and MLR were elevated in the patients with IMNM compared to the HC. The patients with DM showed significantly increased neutrophil percentages, monocyte percentages, monocyte counts, NLR, MLR, and PLR, as well as decreased lymphocyte percentages and counts, compared to the HC. When directly comparing DM and IMNM, the patients with DM had lower lymphocyte percentages and counts, along with higher NLR and MLR. Receiver operating characteristic (ROC) curve analysis revealed that lymphocyte percentages and the MLR had moderate predictive value for differentiating IMNM from DM, with area under the curve (AUC) values of 0.709 and 0.7487, respectively.

**Conclusion:**

RDW and the MLR in IMNM and the NLR, MLR, and PLR in DM represent accessible and cost-effective biomarkers for assessing inflammation. Lymphocyte percentages and the MLR may serve as inexpensive and readily available supplementary markers for distinguishing IMNM from DM.

## 1 Introduction

Idiopathic inflammatory myopathies (IIMs) are a heterogeneous group of autoimmune disorders characterized by proximal weakness and inflammatory infiltrates in biopsied muscle specimens (1). IIMs encompass several clinicopathologic subtypes: dermatomyositis (DM), anti-synthetase syndrome, immune-mediated necrotizing myopathy (IMNM), inclusion body myositis, polymyositis, and overlap myositis (2). IMNM is strongly associated with anti-signal recognition particle (SRP) (3) and anti-3-hydroxy-3-methylglutaryl-coenzyme A reductase (HMGCR) antibodies (4). DM is linked to several autoantibodies, primarily including anti-mitochondrial M2-associated protein (Mi-2), anti-melanoma differentiation-associated protein 5 (MDA5), anti-transcription intermediary factor 1-*γ* (TIF1-γ), anti-nuclear matrix protein 2 (NXP2), and anti-small ubiquitin-like modifier activating enzyme 1 (SAE1) autoantibodies (5). Despite sharing an autoimmune response-mediated muscle damage, DM and IMNM exhibit distinct pathogenetic pathways.

A complete blood routine test is a cost-effective and readily available diagnostic tool. Neutrophils act as key effectors in acute inflammation and contribute to chronic inflammation and adaptive immune responses (6), while lymphocytes are involved in antibody synthesis and immunomodulatory pathways (7). Monocytes, upon tissue recruitment, differentiate into macrophages or dendritic cells to maintain tissue homeostasis or drive inflammatory responses (8). Biomarkers such as the neutrophil-to-lymphocyte ratio (NLR), monocyte-to-lymphocyte ratio (MLR), and platelet-to-lymphocyte ratio (PLR) have been validated as indicators of systemic inflammation in various conditions, including neuromyelitis optica spectrum disorder (9), multiple sclerosis (10, 11), optic neuritis (11), primary Sjogren’s syndrome (12), and Graves’ orbitopathy (13). However, studies exploring the association between blood cell-derived parameters and IMNM/DM remain limited (14, 15). Therefore, this study aimed to evaluate the clinical value of these parameters in an independent cohort of patients with IMNM and DM.

## 2 Methods

### 2.1 Participants

Medical records from Tongji Hospital of Tongji Medical College, Huazhong University of Science and Technology, were reviewed retrospectively between January 2014 and December 2024. Patients with IMNM or DM were included based on the diagnostic criteria from the ENMC International Workshop on Idiopathic Inflammatory Myopathies (16). All patients underwent skeletal muscle biopsies for confirmatory diagnosis. The patients with IMNM were pathologically characterized by muscle fiber necrosis with sparse or absent inflammatory infiltrates. Eligible patients had complete blood count data collected during their first clinical presentation of IIMs, prior to any immunosuppressive therapy. The exclusion criteria included the following: active endocrinopathy, toxic myopathy, infectious myopathy, amyloidosis, a family history of muscular dystrophy or proximal motor neuropathy, and histopathologic features indicative of other IIM subtypes. This study was approved by the Institutional Review Board of Tongji Hospital (IRB ID: TJ-C20121221), and written informed consent was obtained from all participants.

### 2.2 Clinicopathologic data

Demographic characteristics (age at onset and sex), pattern of muscle involvement, muscle strength assessed using the Medical Research Council (MRC) manual muscle testing scale (17), serum creatine kinase (CK) and lactate dehydrogenase (LDH) levels, disease duration, and the presence of interstitial lung disease (ILD) and skin rash were systematically documented. ILD was detected using chest computed tomography. All the patients underwent skeletal muscle biopsies for histopathological analysis. Serial 7 μm-thick frozen sections were stained using routine methods, including hematoxylin–eosin, modified Gomori’s trichrome, acid phosphatase, NADH-tetrazolium reductase, oil red O, myosin ATPase, Sudan black, cytochrome c oxidase, succinate dehydrogenase, and periodic acid-Schiff staining.

Serum samples from the enrolled patients were tested for myositis-specific antibodies (MSAs), myositis-associated antibodies (MAAs), and connective tissue disease-related autoantibodies. The following MSAs and MAAs were evaluated using two commercial semiquantitative line blot assays (D-Tek, Germany; Euroline, Germany): anti-Mi2α and *β*, anti-TIF1γ, anti-MDA5, anti-NXP2, anti-SAE1, anti-Jo1, anti-SRP, anti-HMGCR, anti-PL7, anti-PL12, anti-EJ, anti-OJ, anti-cN-1A, anti-Ku, anti-PMScl100, anti-PMScl75, and anti-Ro52 (18). The following antibodies were tested at the Tongji Hospital Laboratory: anti-nuclear, anti-SSA/Ro60, anti-SSB/La, anti-Sm, anti-RNP, anti-mitochondrial, anti-dsDNA, and rheumatoid factor.

### 2.3 Statistics

Statistical analysis was performed using IBM SPSS Statistics (version 23.0 for Windows; SPSS Inc., Chicago, IL) and GraphPad Prism (version 8.0). To compare data between the groups, the independent Student’s *t*-test was used for continuous variables and Fisher’s exact test was used for categorical data. In the case of non-normally distributed quantitative data, the different groups were compared using the Mann–Whitney U test. Statistical significance was defined at a *p*-value of < 0.05.

## 3 Results

### 3.1 Patient characteristics

During the study period, 44 patients were newly diagnosed with IMNM and 26 patients were diagnosed with DM. Of these, 27 (61.4%) IMNM and 14 (53.8%) DM cases with available pre-treatment complete blood count data were included. Among the patients with IMNM, 23 were anti-SRP antibody-positive, three were anti-HMGCR antibody-positive, and one was seronegative for IMNM. In the DM cohort, six were anti-MDA5 antibody-positive, three were anti-Mi-2 antibody-positive, one was anti-NXP2 antibody-positive, two were anti-TIF1γ antibody-positive, and two were anti-SAE1 antibody-positive. Healthy controls (HC) (*n* = 85) without neuromuscular disorders served as the control group (Table 1).

**Table 1:** Demographic and baseline characteristics of NAM, DM, and HC (Mean ± SD, *p* value).

Items	HC (*n* = 85)	IMNM (*n* = 27)	DM (*n* = 14)	*p*-value for difference IMNM-HC	*p*-value for difference DM-HC	*p*-value for difference IMNM-DM
Demographic						
Age	49 (13, 74)	42.67 ± 16.44	41.57 ± 13.65	0.2681	0.2688	0.8319
Gender	51, 60%	15, 56%	7, 50%	0.8227	0.5632	0.5632
Clinical features						
Muscle weakness	-	26, 96%	13, 93%	-	-	>0.9999
Myalgia	-	7, 25.93%	9, 64.29%	-	-	**0.0229**
Dysphagia	-	7, 25.93%	1, 7.14%	-	-	0.2267
Dyspnea	-	3, 11.11%	0	-	-	0.539
ILD	-	5, 18.52%	2, 14.29%	-	-	>0.9999
Skin rash	-	1, 3.70%	7, 50.00%	-	-	**0.0010**
Muscle strength (MRC)	-	3 (2, 5)	4 (2, 5)	-	-	0.2973
Disease duration, months	-	6.000 (0.5, 36)	2.000 (0.25, 48)	-	-	0.0534
Laboratory findings						
Initial CK, U/L	-	4,509 ± 1873	363 (35, 10,290)	-	-	**0.0035**
Initial LDH, U/L	-	799.4 ± 323.7	419.5 ± 215.7	-	-	**0.0003**

H, healthy control; SD, standard deviation; MRC, Medical Research Council; NAM, necrotizing autoimmune myopathy; DM, dermatomyositis; ILD, interstitial lung disease; LDH, lactate dehydrogenase. Data is not normally distributed and expressed as median with range. NAM group consists of: 23 for anti-SRP-positive NAM, 3 for anti-HMGCR-positive NAM, 1 for seronegative NAM. DM group consist of: 6 for anti-MDA5-positive DM, 3 for anti-Mi-2-positive DM, 1 for anti-NXP2-positive DM, 1 for anti-TIF1γ-positive DM, 2 for anti-SAE1-positive DM, 1 for anti-SRP-positive DM. Boldfaced text denotes *p*-values with *p* < 0.05.

The demographic and clinical characteristics of the patients with IMNM and DM are summarized in Table 1. The median age at onset was 42.67 ± 16.44 years for IMNM and 41.57 ± 13.65 years for DM. Of the 27 patients with IMNM, 26 presented with proximal limb weakness and one was admitted with myalgia without muscle weakness. Proximal limb weakness was observed in 13 of the 14 patients with DM. At initial presentation, the patients with DM (9/14) more frequently reported myalgia and skin rash (*p* = 0.0010). The levels of serum CK and LDH at onset were significantly lower in the DM group than in the IMNM group (*p* = 0.0035; *p* = 0.0003). The patients with DM tended to have a shorter disease duration, although the difference did not reach significance.

### 3.2 Comparisons of complete blood cell-derived parameters between the groups

The patients with IMNM exhibited significantly higher monocyte counts, red blood cell distribution width (RDW), and MLR compared to the HC. The patients with DM had elevated neutrophil and monocyte percentages and counts, reduced lymphocyte percentages and counts, and higher NLR, MLR, and PLR than the HC. No significant differences in the levels of leucocytes, eosinophils, basophils, platelets, platelet distribution width, or mean platelet volume were observed between the HC and either patient group (Table 2).

**Figure 1:**
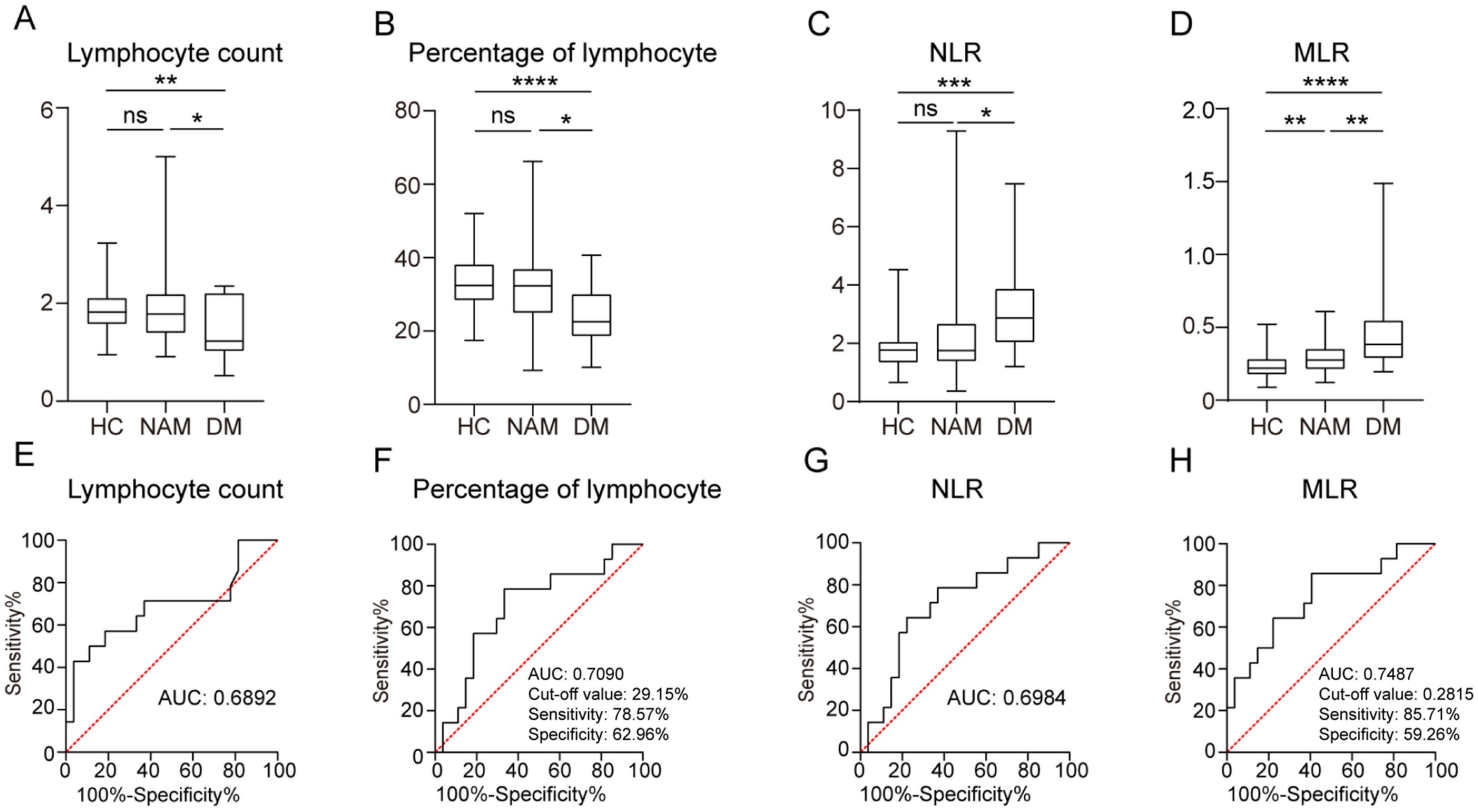
Peripheral lymphocyte count, lymphocyte percentage, NLR, and MLR levels differentiate the patients with IMNM and DM. **(A–D)** Circulating lymphocyte count, lymphocyte percentage, NLR, and MLR levels in the patients with IMNM, patients with DM, and HC. *p < 0.05; **p < 0.01; ***p < 0.001; ****p < 0.0001. **(E–H)** Lymphocyte count, lymphocyte percentage, NLR, and MLR levels in receiver operating characteristic models. The corresponding area under the curve values were 0.6892, 0.7090, 0.6984, and 0.7487, respectively.

When comparing DM and IMNM directly, the patients with DM showed substantially lower lymphocyte percentages and counts, along with higher NLR and MLR (Table 2, Figure 1). Receiver operating characteristic (ROC) curve analysis revealed moderate diagnostic utility for differentiating IMNM from DM. The area under the curve (AUC) values were 0.6892 for lymphocyte percentage, 0.7090 for lymphocyte count, 0.6984 for the NLR, and 0.7487 for the MLR (Figure 1).

**Table 2:** Blood parameters of patients with IMNM and DM (Mean ± SD, p value).

Laboratory Findings	Normal range	HC (*n* = 85)	IMNM (*n* = 27)	DM (*n* = 14)	*p*-value for difference IMNM-HC	*p*-value for difference DM-HC	*p*-value for difference IMNM-DM
Leucocytes, 10^9^ per L	3.5–9.5	5.380 (3.04, 10)	5.9503 (3.630, 13.59)	5.310 (4.03, 12.74)	0.085	0.6782	0.5362
Neutrophils, %	40.0–75.0	55.84 ± 7.757	57.38 ± 13.38	64.21 ± 9.888	0.4599	**0.0005**	0.1001
Neutrophils, 10^9^ per L	1.8–6.3	2.950 (1.27, 7.2)	3.300 (1.190, 11.79)	3.700 (1.87, 9.38)	0.2202	0.1026	0.6009
Lymphocytes, %	20.0–50.0	33.82 ± 7.293	31.51 ± 11.63	23.86 ± 8.949	0.2228	**<0.0001**	**0.038**
Lymphocytes, 10^9^ per L	1.1–3.2	1.84 ± 0.422	1.780 (0.91, 5)	1.424 ± 0.6034	0.8964	**0.0098**	**0.0493**
Monocytes, %	3.0–10.0	7.534 ± 2.004	8.259 ± 2.088	8.300 ± 3.174 (6.2, 15.7)	0.0806	**0.0324**	0.4751
Monocytes, 10^9^ per L	0.1–0.6	0.3900 (0.14, 0.93)	0.4700 (0.32, 0.86)	0.5200 (0.32, 1.92)	**0.0032**	**0.0132**	0.6389
Eosinophils, %	0.4–8.0	2.100 (0.2, 7.5)	2.000 (0, 6.5)	1.971 ± 1.574	0.875	0.2936	0.4583
Eosinophils, 10^9^ per L	0.02–0.52	0.1100 (0.01, 1)	0.1000 (0, 0.37)	0.1221 ± 0.1025	0.942	0.3926	0.4096
Basophils, %	0.0–1.0	0.4000 ± 0.2443	0.3000 (0, 1.2)	0.3429 ± 0.2623	0.1957	0.2342	0.8536
Basophils, 10^9^ per L	0.00–0.10	0.02000 (0, 0.09)	0.02000 (0, 0.5)	0.02000 (0, 0.07)	0.5715	0.3679	0.7115
Platelet, 10^9^ per L	100–300	211.3 ± 51.79	231.2 ± 70.34	216.9 ± 74.85	0.1151	0.7248	0.5505
RBC distribution (CV)	<14.9	12.50 (11.4, 14.8)	13.60 (9.9, 16.9)	12.75 (11.9, 15.6)	**<0.0001**	0.0672	0.3292
RBC distribution (SD)	39.0–46.0	42.17 ± 2.65	46.00 (10.8, 55.8)	43.61 ± 3.393	**<0.0001**	0.1259	0.058
Platelet distribution width	9.0–17.0	13.79 ± 2.354	14.51 ± 2.802	12.50 (9, 24.9)	0.3631	0.3274	0.1099
Mean platelet volume	8.0–15.0	11.27 ± 1.079	11.40 (6.6, 46.4)	10.69 ± 1.682	0.6984	0.1112	0.1481
NLR	-	1.770 (0.66, 4.53)	1.753 (0.36, 9.283)	2.867 (1.201, 7.481)	0.4002	**0.0003**	**0.0395**
MLR	-	0.2200 (0.09, 0.52)	0.2761 (0.122, 0.6099)	0.3830 (0.1964, 1.488)	**0.0084**	**<0.0001**	**0.0089**
PLR	-	118.1 ± 29.19	119.8 (42.4, 300)	148.5 (81.25, 455.8)	0.4156	**0.0042**	0.0671

HC, healthy control; NLR, neutrophil-to-lymphocyte ratio; MLR, monocyte-to-lymphocyte ratio; PLR, platelet-to-lymphocyte ratio; SD, standard deviation; CV, coefficient of variation; NAM, necrotizing autoimmune myopathy; DM, dermatomyositis. Data is not normally distributed and expressed as median with range. NAM group consists of: 23 for anti-SRP-positive NAM, 3 for anti-HMGCR-positive NAM, 1 for Seronegative NAM. DM group consist of: 6 for anti-MDA5-positive DM, 3 for anti-Mi-2-positive DM, 1 for anti-NXP2-positive DM, 1 for anti-TIF1γ-positive DM, 2 for anti-SAE1-positive DM, 1 for anti-SRP-positive DM. p < 0.05 is statistically significant. Boldfaced text denotes *p*-values with *p* < 0.05.

## 4 Discussion

In agreement with previous studies (16), our study demonstrated that the patients with IMNM exhibited symmetrical, proximal limb muscle weakness and significantly elevated levels of serum CK and LDH. In contrast, myalgia and cutaneous manifestations were more prevalent in the patients with DM compared to those with IMNM.

The NLR, MLR, and PLR are rapid, cost-effective, and reliable markers of inflammation in many autoimmune diseases (9, 12, 13, 19). In the current study, the patients with DM displayed elevated neutrophil percentages, reduced lymphocyte percentages and counts, elevated monocyte percentages and counts, and higher NLR, MLR, and PLR compared to the HC. These findings are consistent with those of previous studies highlighting the roles of the NLR and PLR in DM pathogenesis (14, 20). The exact mechanisms responsible for the increased peripheral neutrophils and monocytes in DM remain elusive, although several studies have indicated that dysregulated neutrophil function may contribute to the immunopathogenesis of DM (21–23).

RDW has been widely studied in the etiology of anemia and is recognized as an effective diagnostic and prognostic index in autoimmune disorders (12), cancer (24), and cardiovascular diseases (25). In our analysis, significantly higher RDW was observed in the IMNM group compared to the HC. A previous study linked elevated RDW to disease activity in polymyositis and DM (14, 15). Conversely, our data did not demonstrate increased RDW in DM, which may be attributed to the relatively small sample size. Nevertheless, further prospective studies with a larger sample size are clearly warranted to clarify the role of RDW in IIMs.

In our study, the elevated MLR in IMNM was likely driven by increased monocyte counts, as no statistical difference in lymphocyte counts was observed compared to the HC. Previous investigations have demonstrated that type 1 helper T cell-driven macrophages are the dominant mononuclear cellular infiltrate in IMNM (26, 27). This suggests that a robust type 1 helper T cell-mediated immune response may promote the production of monocytes/macrophages in IMNM. While the role of the adaptive immune system, including autoantibodies and complement pathways, in IMNM pathogenesis has been extensively studied (28, 29), the observation of peripheral monocytosis in IMNM adds to the understanding of the lesser-explored role of the innate immune system in this disease.

When comparing DM to IMNM, the elevated NLR and MLR in DM were largely driven by the decreased lymphocyte counts, as no significant intergroup differences were observed in neutrophil counts or monocyte counts. Lymphocyte dysfunction, including humoral immunity mediated by B lymphocytes and the activation and infiltration of T helper cells, is a critical pathophysiologic mechanism in DM (30). In contrast to the pathogenic features of DM, IMNM is characterized by a type 1 helper T cell/classically activated macrophage M1 response within muscle tissues (26). Our findings imply distinct roles for lymphocytes in these two diseases. The chemotactic recruitment of lymphocytes into damaged tissues may explain the peripheral lymphocytopenia observed in DM, a hypothesis that partially accounts for our observed reduction in circulating lymphocytes. However, further investigations into the immune mechanisms of DM are needed to clarify the details of circulating lymphocyte decreases and subtype alterations.

This study has several limitations. First, the small sample size from a single medical center and the retrospective nature of the study suggest that biases are inevitable. While the preliminary predictive values suggest utility for the MLR and lymphocyte percentage, validation in larger, multicenter cohorts is essential to confirm cutoff values and account for population heterogeneity. These efforts are underway and will strengthen the biomarkers’ clinical translation. Second, our results may not be generalizable to other racial populations, as all participants were of Han Chinese ethnicity. Lastly, the absence of detailed inflammatory cell profiling in muscle biopsies represents a mechanistic limitation. While our prior work showed macrophage-dominant infiltrates in IMNM (31), future studies should explore neutrophil and lymphocyte subsets to better contextualize systemic biomarker ratios in relation to local tissue immunity.

In conclusion, our study compared peripheral blood parameters among patients with DM, patients with IMNM, and HC. We concluded that RDW and the MLR in IMNM, as well as the NLR, MLR, and PLR in DM, may serve as easily available and cost-effective tools in the evaluation of inflammation. Furthermore, lymphocyte percentages and the MLR may help differentiate IMNM from DM.

## Data Availability

The raw data supporting the conclusions of this article will be made available by the authors, without undue reservation.
